# Shield Maidens, Fashy Femmes, and TradWives: Feminism, Patriarchy, and Right-Wing Populism

**DOI:** 10.3389/fsoc.2020.619572

**Published:** 2020-12-23

**Authors:** Nancy S. Love

**Affiliations:** Department of Government and Justice Studies, Appalachian State University, Boone, NC, United States

**Keywords:** white supremacy, alt right, patriarchy, populism, feminism, intersectionality

## Abstract

Although media images typically present the alt right as a “manosphere,” white women continue to participate actively in white supremacist movements. Alt right women's presence as “shield maidens,” “fashy femmes,” and “trad wives” serves to soften and normalize white supremacy, often in ironic and insidious ways. In this essay, I examine the continued investment of white women in these traditional sex/gender roles espoused by the alt right. While feminism has done much to liberate women, I conclude that the images of women as Moms circulating in mainstream politics today suggest that white supremacy and white women's complicity in it has yet to be overcome.

Media images of the 2017 “Unite the Right” rally in Charlottesville, Virginia featuring angry white men chanting and marching with tiki torches confirmed public perceptions of the alt right as a “manosphere.” The alt right *is* hypermasculine, misogynist, and antifeminist. It has formed alliances with involuntary celibates (incels), men's rights advocates (MRAs), and pick up artists (PUAs). Its “thought leaders” argue against higher education, professional careers, reproductive rights, and voting rights for women (Hayden, 2017; Center on Extremism Report, 2018). The alt right opposes “women's liberation” because it gives women choices that make it less likely that we will “get married, have children, and perpetuate the white race” (Center on Extremism Report, 2018, p. 7). Its members call liberated women “thots,” which means “that ho over there,” and celebrate the femininity and fertility of women who accept their traditional sex/gender roles, calling them as “tradhots” (Center on Extremism Report, 2018, p. 6–7). In short, the alt right would return white women to our presumably natural biological roles as wives and mothers for the white race.

This dominant image of the alt right as a “manosphere,” however accurate it may be, obscures the long history of white women's participation in white supremacy. White women were active in the Ku Klux Klan, the American Nazi Party, and more recently, have joined neo-Nazi groups, such as the Aryan Nation, National Vanguard, White Aryan Resistance, and now the alt right (Blee, 1992, 2003; Schabner, 2006; Love, 2016). In order to understand more fully the roles of white women – and men – in white supremacy an intersectional analysis is needed. According to Patricia Hill Collins, “As opposed to examining gender, race, and class, and nation as separate systems of oppression, intersectionality explores how these systems mutually construct one another, or, in the words of Black British sociologist Stuart Hall, how they ‘articulate' with one another” (Collins, 1998, p. 63). Collins' intersectional analyses stress gender, nation, and race, due to their prominence in constructions of the United States as a racialized “family writ large” (1998, p. 64). Black female scholars developed intersectionality to analyze the multiple forms of oppression experienced by Black women (Hancock, 2016). However, I argue elsewhere that it can also unpack relations of white power and privilege (Love, 2012). In the process, intersectional analyses highlight the linkages of class to gender, nation, and race in constructions of the American worker as white and male (Roediger, 2007).

Women in white supremacist movements, including the alt right, typically serve as auxiliaries rather than leaders. This partly explains why women's participation receives less media and scholarly attention. As movement auxiliaries, white women's role is to soften and normalize white supremacy, earning them the label “shield maidens.” For example, a former white supremacist, Samantha, who organized the women's group “Warriors for the Home Front” for the alt right Identity Europa, booked the log cabin accommodations at a nearby winery for alt right leaders after Charlottesville 1.0, a pilot rally at the Robert E. Lee monument. She explains: “I thought it would be funny if [anti-fascist activists] wanted to chase us out of town. you know like, 'Oh these big scary Nazis retreated to a vineyard.' I thought it would be profoundly ironic” (Reeve, 2019). Like Samantha, other alt right organizers embrace ambiguous and ironic representations of white supremacy (Wilson, 2017). White women also shield white supremacy in less subtle and more traditional ways, representing their roles as community service and social welfare. Women in white supremacist groups have organized church socials, Klan picnics, and more recently, charity fundraisers and white nationalist online dating sites.

Perhaps the best example of alt right views on traditional sex/gender roles is the TradWives, a group of white nationalist “mommy vloggers,” who promote the “virtues of staying at home, submitting to male leadership, bearing lots of children” (Kelly, 2018). These women extol a 50 s escapist fantasy of “chastity, marriage, motherhood,” a fantasy that Betty Friedan famously exposed as “magical thinking” in *The Feminine Mystique* (Friedan, 1963). TradWives construct a “hyperfeminine aesthetic” in order to “mask the authoritarianism of their ideology” (Kelly, 2018). Often women only face the reality of white supremacist misogyny when they, like Samantha, must risk their – and sometimes their children's – lives to leave the movement (Zia, 1991; Reeve, 2019). Some alt right women further weaponize femininity against feminism with *Cosmopolitan*-like promotions of fashion and makeup, earning them the label, “fashy femmes.” Wolfie James, wife of the alleged white nationalist, Matthew Gebert, exemplifies this approach. Of alt-right men, James says, “the masculinity they exude is positively intoxicating” (James, 2017; Hesse, 2019). James argues that “although men are better suited to the cause” given their greater physical strength and capacity for violence, it is women who can “boost it to the next level” (Hesse, 2019).

These alt right women claim feminism has failed white women, robbing us of the opportunity to have a male provider, a happy family, and a nice home. According to this narrative, the #MeToo movement only confirms the dangerous world feminism has created for women, a world where men no longer respect us for our femininity and fertility and, hence, feel free to assault, harass, and rape us. According to one teen, in this brave new feminist world, “traditionalism does ‘what feminism is supposed to do' in preventing women from being made into ‘sex objects' and treated ‘like a whore”' (Smith, 2017). This narrative also laments how white men have been robbed of their rightful status; their jobs and roles have been taken by women, people of color, and immigrants in the workforce. Some incels and men's rights activists, who argue that men are entitled to sex with women, claim that refusing them is “reverse rape” and call for their own #MeToo movement (Center on Extremism Report, 2018, p. 12). In “The Problem of Surplus White Men,” John Feffer concludes that “white men who are all revved up with nowhere to go pose the greatest challenge to democracy in America” (Feffer, 2020). Feffer notes that many of these men are Trump supporters. These white men and women provide fertile ground for an anti-modern populist mobilization (Kelly, 2018). Following Trump's 2016 victory, Lana Lokteff, another alt right organizer, said: “Our enemies have become so arrogant that they count on our silence….When women get involved, a movement becomes a serious threat” (Smith, 2017).

Of course, mothers are also politically active on the political left, and progressive movements also use resentment to mobilize supporters, though more reluctantly than the populist right (Dolgert, 2016). Further, women have long had primary responsibility for “care work” across the political spectrum. Silvia Federici writes, “‘Reproduction' has two sides, in contradiction with each other. On the one hand it reproduces us as people, and on the other it preproduces us as exploitable workers” (Federici and Sitrin, 2016). This contradiction means that women, especially women of color and their children, disproportionately experience the effects of poverty under capitalism, an oppressive reality that Black Lives Matter protests of systemic racism confront. Yet mainstream media only featured women's presence in the Portland, Oregon protests when a multiracial organized group of mothers arrived. Wearing bike helmets and face masks, they formed a “Wall of Moms” and chanted “Moms Are Here; Feds Stay Clear.” Their actions reinvoked the Argentinian Madres de Plaza de Mayo who protested the “disappearances” of their children in the 1970's (Barajes, 2020). They honored women's power to bring life – literal and metaphorical – into the world, and highlighted the connections between justice, rights, and care (Federici and Sitrin, 2016; Tronto, 2020).

Yet this imagery of women as mothers and activists across the political spectrum is troubling in many respects. Why did it take the arrival of white Moms for the mainstream media to portray the Black Lives Matter protestors as mothers fearing and fighting for their children? After all, Black Lives Matter was founded by three women, Patrisse Cullors, Alicia Garza, and Opal Tometi in 2013 after George Zimmerman was acquitted of the murder of Trayvon Martin. To reinvoke Collins, is our dominant image of the American “national family” still all too white? Further, in our current pandemic economy with its “stay at home” and “safer at home” orders, domestic violence has increased, women are disproportionately tasked with childcare and home schooling, and the needs of women of color, single women, many of them elderly, and single working mothers, are minimized or bypassed. It still and again seems women can have a child or a career, but not both (Perelman, 2020). What hierarchies of race, gender, and class are reproduced here, and for whom?

I am not equating the pandemic return to traditional sex/gender roles or the powerful presence of mothers in Black Lives Matter protests with recent increases in white supremacist racism and misogyny. Yet sometimes an extreme can illuminate the norm. These images of women as mothers show that patriarchy runs deeper in American society than the polarized politics of right and left. They also remind us that because patriarchy is intersectional, resistance to it must also be. White women, who were once the slave mistresses of plantation households, have continued to normalize white supremacy, to shield it with their delusions of domesticity, purity, and vulnerability (Glymph, 2008, Smith, 2018 [interview with Linda Gordon]). According to Barbara Smith, “‘systemic racism' connotes the pervasiveness of racial oppression, but white supremacy goes further by indicating that there is a rigid nexus of power that protects and enforces it” (Smith, 2020). Men and Moms – perhaps these images of masculinity and femininity circulating today can remind white feminists that white supremacy is a power nexus we have yet to dismantle. If further proof is needed, 53% of white women voted for Trump and 92% of black women voted for Hillary in 2016. Although pre-election polls suggested those numbers might change in 2020, the majority of white women – and men – again voted for Trump (Schwadron, 2020). At this writing, Biden's presidential victory is not yet certified.

## Author Contributions

The author confirms being the sole contributor of this work and has approved it for publication.

## Conflict of Interest

The author declares that the research was conducted in the absence of any commercial or financial relationships that could be construed as a potential conflict of interest.
